# Combination of High-Dose Intravenous Immunoglobulins and Itraconozole in Treating Chronic Mycotic Demyelinating Optic Neuritis

**DOI:** 10.1100/tsw.2003.49

**Published:** 2003-08-02

**Authors:** Andrew W. Campbell, Ebere C. Anyanwu, Aristo Vojdani

**Affiliations:** Medical Center for Immune, Environmental and Toxic Disorders, 25010 Oakhurst, Suite 200, Spring, Texas 77386, USA

**Keywords:** intravenous immunoglobulins, optic neuritis, neuron specific antigens, United States, visual evoked potentials, fungal infections, drug treatment

## Abstract

Mycotic demyelinating optic neuritis is a neurological disorder of the visual system caused by mycotoxins released by indoor toxic molds. Although the health effect of indoor toxic mold on the population worldwide is now one of the “emerging diseases”, its involvement in chronic demyelinating optic neuritis has not been reported. Most of the neurological and immunologic abnormalities associated with toxic mold mycotoxins are very difficult to treat successfully, especially neural demyelination of the central and peripheral nervous systems. This paper presents the case of a 42-year-old white female, in whom chronic demyelinating optic neuritis with persistent visual defects due to chronic exposure to toxic molds was diagnosed at the age of 34 years. In spite of all the therapeutic services given to her for over 8 years, her illness persisted and was difficult to treat. However, we successfully treated her with a combination of intravenous immune globulin (IVIG) and itraconozole (Sporanox) when all other treatment modalities failed. This is probably the first report where persistent toxic mold-induced neurological and immunologic disorders were successfully treated with a combination of itraconozole and IVIG.

## INTRODUCTION

Mycotic demyelinating optic neuritis is a neurological disorder of the visual system caused by mycotoxins released by toxic molds. It is characterized by gross demyelination of the optic nerves and is accompanied by discomfort in or around the eye with increase in eye blinking. Like other types of optic neuritis, the signs and symptoms are variable: unilateral blurring of vision, pain in the eye at rest, remission and paracentral scotoma, attacks of double vision, trigeminal neuralgia, and sometimes unintentional tremor. Extraocular muscles may be affected, resulting in the disturbances of conjugate movement; gaze palsy; horizontal, mixed, or vertical nystagmus; and abnormal light-blink reflex[1]. The resulting demyelination leads to general cell losses with the consequent reduction in cell density, axonal damage, and ganglion cell deaths, thereby affecting the visual transmission. Because of the propensity to produce extremely potent lipid-soluble mycotoxins that are readily absorbed by the intestinal lining, airways, and skin[2], toxic mold exposures result in adverse health consequences to the exposed individuals.

Previously, we reported some of the neurological and immunologic manifestations of indoor toxic mold exposure[3]. In the present study, we report the case of a 42-year-old white female, who was chronically exposed to indoor toxic mold. She presented with chronic mycotic demyelinating optic neuritis and was treated with a combination of IVIG and itraconozole.

## CASE REPORT

A 42-year-old white female attended our Center for clinical evaluation and treatment presenting with visual disturbances including unilateral blurring of vision, pain in the eye at rest, remission and attacks of double vision, abnormal light-blink reflex, and sometimes unintentional tremor. Extraocular muscles were affected, resulting in the disturbances of conjugate movement and gaze. She complained of dizziness, numbness and tingling in the extremities, fatigue, memory loss, sleep disturbances, headaches, decreased libido, muscle and joint aches, night sweats, sores that would not heal, bruising easily, facial twitching, and recurrent flu-like illnesses.

Her family had moved into an old stucco home in the Woodlands area of Texas in 1993. In the last 5 years she has noticed more headaches, colds, and illnesses than previously. However, in the last 3 years she has had problems with her throat and her voice has also changed. Mycological testing of her house found *Stachybotris* sp., *Aspergillus* sp., and *Penicillium* sp., as well as numerous other fungal species.

Her physical examination further revealed fairly intact tympanic membranes with abnormal light reflex, her throat was dry, and her pupils were very sluggish. Her neck was supple and her lungs were fairly clear. Auscultation of her heart revealed irregular rate and rhythm with murmurs. There was axillary lymphadenopathy and her abdomen was soft without masses or hepatomegaly. There was no inguinal lymphadenopathy; her back was normal with no costovertebral angle pain. She had full range of motion without clubbing or edema. Neurological examination was abnormal with hyper-reflexic deep tendon reflexes in all four extremities. Her skin was "red-rashy" and dry. She was previously placed on steroids, Claritin (10 mg QD), Zantac (150 mg BID), and Ambien (10 mg QHS) medications, but was not responsive to the treatment. Rather, her condition got worse with additional allergic reactions.

Immunologic investigations revealed abnormal antibodies to *Penicillium notatum, Phoma herbarium, Rhodotorula glutinis, Epicoccum nigerium, Geotrichum candidum*, *Candida*, and *Stachybotrys* at chronic values. Enzyme-linked immunosorbent assay (ELISA) of the patient's sera revealed multiple neuroimmunologic abnormalities, including elevation of antibodies (IgG, IgM, IgA) against different neuron-specific antigens. These antigens include myelin basic protein (MBP), myelin-associated glycolipids (MAG), ganglioside (GM1) sulfatide, and chondroitin sulfate.

We carried out a series of neurophysiological and immunologic tests and found that her EEG was slightly abnormal especially in the occipito-parietal region of the brain. Visual evoked potential (VEP) had increased latencies in both eyes with prolonged latencies and diminished amplitudes of P100. The morphology of the N75 was diminished (Fig. 1).

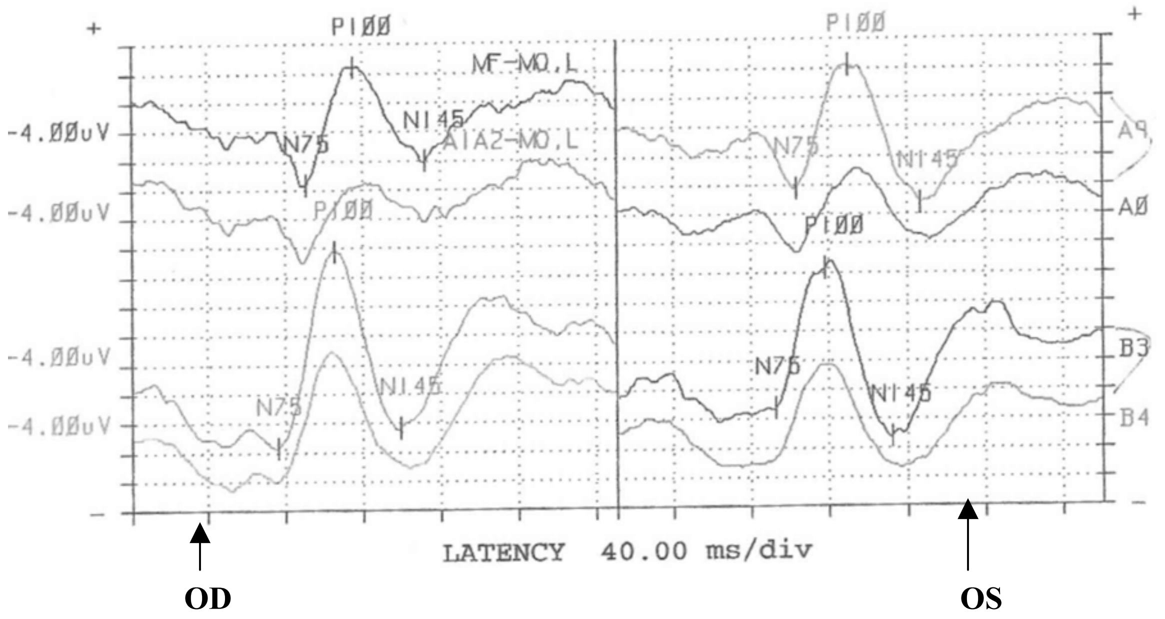

**FIGURE 1.** Pattern reversal VEPs in the left column are responses to 15’ checks and in the right column are responses to 31’ checks. The P100 is delayed with prolonged average latency of over 80 msec. Note the destructive effect of the disorder (retinopathy) on the N75. (As = 15’ checks; Bs = 31’ checks).

The patient showed increased interocular latency difference too. The brainstem auditory evoked response (BAER) was abnormal. Spirometry testing demonstrated borderline obstruction. Sensory nerve conduction threshold (CPT) tests taken from six sites were abnormal and the bilateral measurements obtained from the trigeminal nerve anterior to the tragus gave grades of 9.00 and 9.37 on the left and right sides, respectively, indicating severe hypoesthetic conditions. Bilateral measurements from the median nerve on the index finger; C7 and the deep and superficial peroneal nerves on toe 1; L4, L5 were normal.

The patient was placed on Sporanox 200 mg po BID. In addition she was started on IVIG Gamimune N 10% 0.4 GM/KG/Dose 1-x wk × 12 weeks. At the end of 12 treatments of IVIG, she felt much better. Her energy was restored and the symptoms that were persistent and severe, disappeared. On re-examination, neuroimmunologic abnormalities improved tremendously although, her serology continued to show slightly elevated T- and B-cell function and there were still myelin basic protein antibodies present. All the evoked responses were significantly and positively modified after treatment with IVIG except the VEP showed slight abnormalities (see Fig. 2).

She was advised to continue with IVIG at the same dose in order for her to continue to improve and regain her former health since the effect of this therapy is, however, transient and improvement has to be maintained with periodic infusions[4,5]. She noted that there has been some great relief especially since she moved out of their home.

## DISCUSSION

The paper presented the case of a 42-year-old female diagnosed as having demyelinating optic neuritis due to chronic exposures to toxic molds. The growth of toxic molds is pervasive throughout the outdoor environment, however, given the proper growth conditions, they proliferate in the indoor environmental settings. Toxic molds readily enter indoor environments

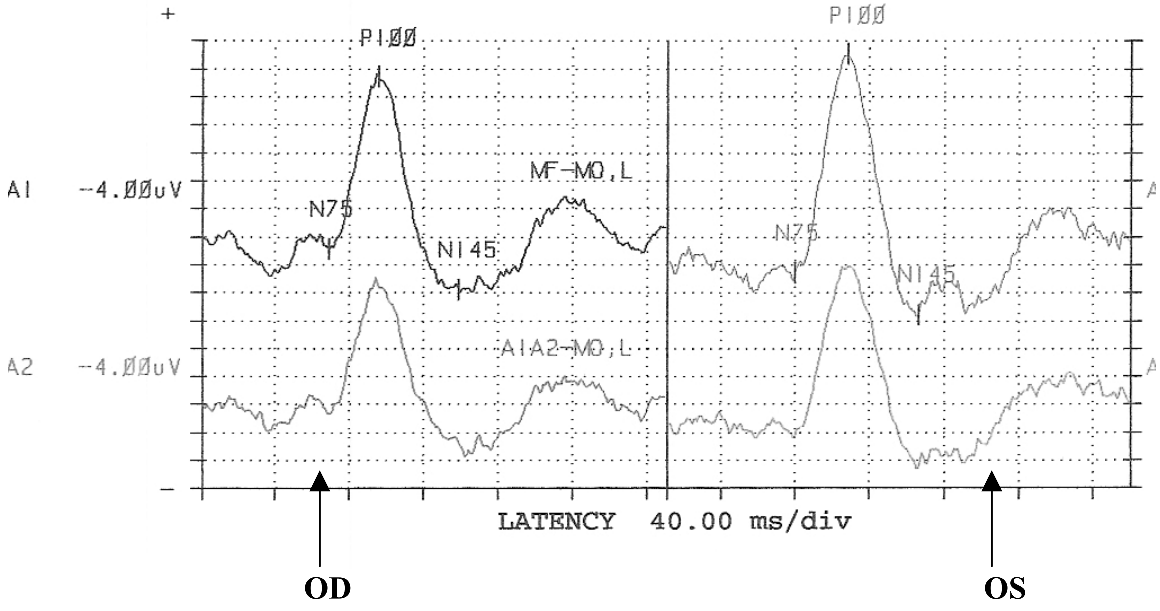

**FIGURE 2.** Although the morphology of the VEP improved in both responses to 15' checks and 31' checks after treatment with IVIG, observe that the P100 latencies in both cases were prolonged (A1 = 15' checks; A2 = 31' checks).

by circulating through doorways, windows, heating, ventilation systems, and air conditioning systems. Because most people spend part of their time indoors, they are exposed to the toxic molds[6,7]. Toxic molds release mycotoxins that affect both cellular and humoral immunity leading to diverse and powerful biological effects. Some are carcinogenic, mutagenic, teratogenic, estrogenic, hemorrhagic, immunotoxic, nephrotoxic, hepatotoxic, dermotoxic, and neurotoxic[8].

In most cases of optic neuritis, there are delays of WF responses range of 10–100 msecs with mean values of 30–40 msecs. Also, as a result of the presence of demyelination, ipsilateral HF components of the VEP appear to show differential latency changes with earlier waves (e.g., N75 and the "leading descending limb" of the P100) showing less marked delays than the later potentials (e.g., the "trailing ascending limb" of the P100 and the N145 wave)[9]. It is shown that pattern reversal stimulation was an effective diagnostic technique in identifying delays in VEP due to demyelination[10,11,12]. This effectiveness has been consistent with and confirmed by our findings. The occurrence of demyelinating optic neuritis in our patient is correlated with VEP changes and the presence of antibodies against neuron-specific mold antigens. This is consistent with the high frequency of abnormal VEPs and antibodies against glycolipids found in patients with CNS and peripheral neural demyelinating lesions[13]. The VEP abnormalities in our patient may also be explained by the susceptibility to immune-mediated damage of the optic nerve.

Furthermore, the association of demyelinating neuropathy with monoclonal gammopathy has been known for several years, and antibodies, mostly IgM, to the gangliosides GM1 are frequently detected in patients' sera, helping in the diagnosis of some neuropathic diseases[14]. Antisulfatide IgM antibodies have been recently associated with neuropathy but the clinical and electrophysiological correlations of this reactivity remains unclear[15]. However, it was shown that patients with high antisulfatide IgM titers had a chronic, dysimmune, mostly sensorimotor neuropathy that was associated with IgM monoclonal gammopathy[16]. These findings were consistent with the electrophysiological and morphological studies and were consistent with a predominantly demyelinating neuropathy frequently associated with prominent axonal loss. Many data point to a pathogenetic role for IgM antibodies to the myelin-associated glycoprotein (MAG)

in the neuropathy associated with IgM monoclonal gammopathy, supporting the use of immune therapies in affected patients[17,18,19].

The presence of high anti-MAG IgM is correlated with the presence of the demyelinating optic neuritis and the development of symptomatic neuropathy supports its pathogenetic role in the optic neuritis. The role of IVIG in treating a variety of diseases is controversial. The decision to use doses was based on clinical studies that demonstrated the efficacy of immunoglobulins treatment in a number of autoimmune and inflammatory conditions[20]. A high dose of IVIG induced a relatively small but long-lasting reduction of autoantibody levels by accelerated IgG clearance. This mechanism has clinical relevance in the sense that it can fully explain the gradual decrease in autoantibody levels observed in several patient studies. However, in some clinical studies, larger or more rapid effects have been observed that cannot be explained by accelerated clearance. Hence, IVIG can also reduce autoantibody levels through mechanisms such as down-regulation of antibody production or neutralization by anti-idiotypic antibodies[20]. For chronic immune-mediated neuropathies such as demyelinating optic neuritis, the diagnosis is made on clinical, electrophysiological, and sometimes immunochemical and pathological criteria[21] and the treatments are sometimes prolonged. As we have observed in our patient, the efficacy of short-term treatments with IVIG depended on the type of the polyneuropathy, but for conditions such as demyelinating optic neuritis, high-dose IVIG may sometimes lead to continuous improvement. Neurophysiological studies are essential for the proper diagnosis, disclosing the presence of conspicuous alterations of the nerve conduction that are mainly of demyelinating type and occur exclusively in the motor fibers. High titers of IgM antibodies, mainly anti-GM1, are frequently observed although their pathogenetic significance has not still been completely established. In many cases, even in those with very prolonged evolutions, treatment with high doses of parenteral immunoglobulins has been effective[22].

## CONCLUSION

Based on the successful treatment of chronic mycotic demyelinating optic neuritis, and with evidence from the literature reviews, high-dose IVIG has emerged as an important therapy for various neurologic diseases. Although very little is currently known about the ramifications and successful treatment of chronic mycosis-related demyelinating optic neuritis, however, the general consensus of treating chronic demyelinating neuropathy showed that about 30% of patients do not respond to any of known conventional procedures[23]. Nevertheless, for our patient, the use of a combination of IVIG and Sporanox has proved relatively more effective. This result outcome supports at least in part, the significance of IVIG in mycotic neuropathy associated with IgG monoclonal gammopathy[24]. Apparently, we believe that IVIG treatment was associated with her improvement in visual function.

## BIOSKETCH

**Andrew W. Campbell** is the Medical Director and Chief Executive of the Medical Center for Immune and Toxic Disorders, Houston, TX. Dr. Campbell is an expert in chemical and immunotoxicology.

**Ebere C. Anyanwu** is a Clinical Neurophysiologist and the Director of Cahers Neurosciences Research Inc., Houston. Dr. Anyanwu is an Adjunct Professor of Anatomy and Physiology at the North Harris and Montgomery Colleges in Houston and The Woodlands, respectively. Dr. Anyanwu is also a Researcher at the Biomarkers Research Laboratory, Department of Environmental Chemistry and Toxicology, Texas Southern University, Houston.

**Aristo Vojdani** is Professor of Immunochemistry and the Chief Executive of the Immunosciences Laboratory, Inc., Beverly Hills, CA.
